# Supplementary material for: Angelica sinensis polysaccharide as potential protectants against recurrent spontaneous abortion: focus on autophagy regulation
Source: Front Med (Lausanne). 2025 Jan 15;12:1522503. doi: 10.3389/fmed.2025.1522503 (PMC11774876; doi:10.3389/fmed.2025.1522503)
Supplement: Supplementary material S1 — The main instruments used during the LC-MS process, along with their models/specifications and manufacturers. [file Supplementary_file_1.zip › Metabolomics sequencing data FC1.2/4.多元统计分析/其他图形/Splot-OPLS-DA(Exposure_Ctrl).pdf]

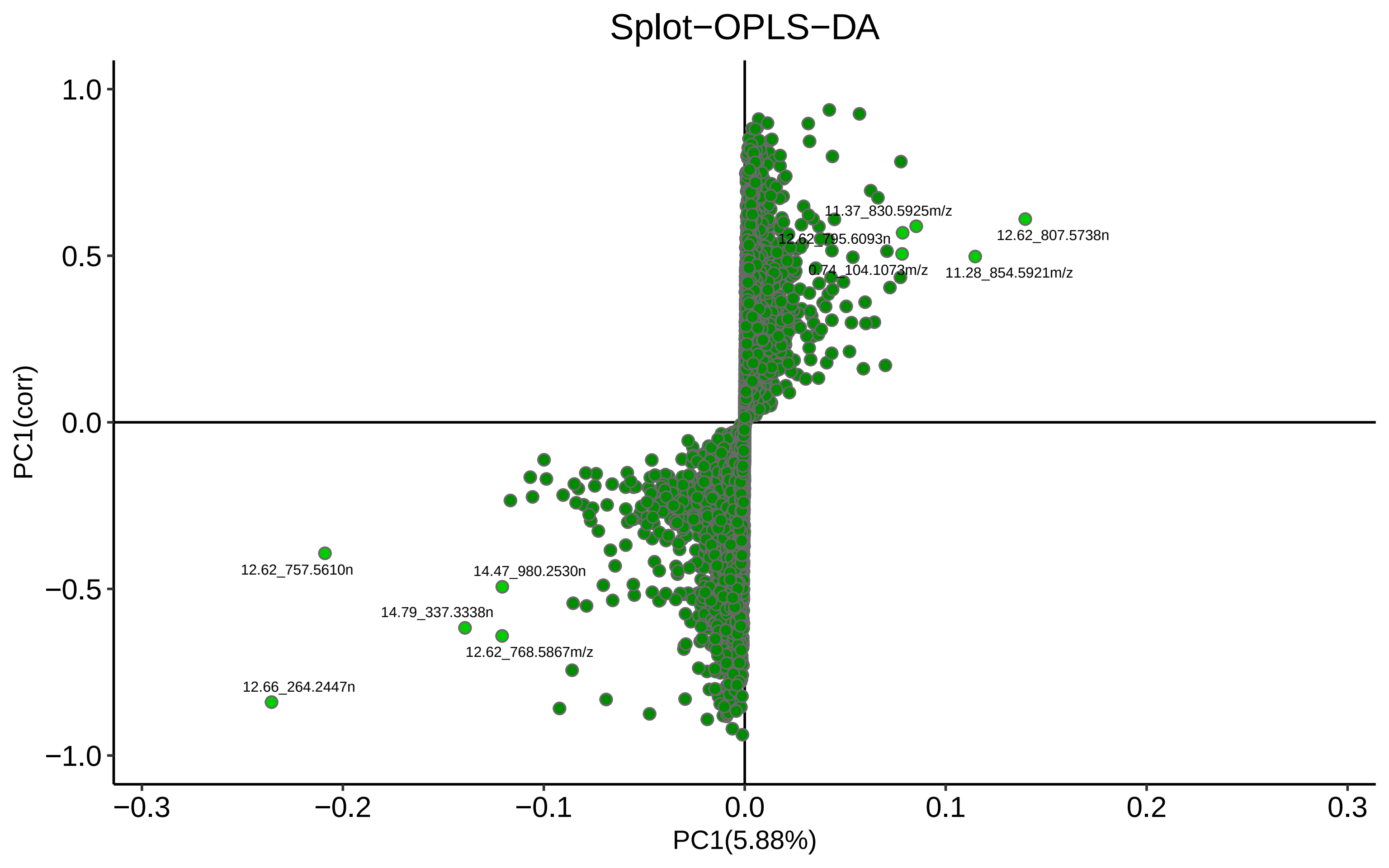

Splot−OPLS−DA
PC1(corr)
PC1(5.88%)
1.0
0.5
0.0
−0.5
−1.0
−0.3
−0.2
−0.1
0.0
0.1
0.2
0.3
11.37_830.5925m/z
12.62_807.5738n
12.62_795.6093n
0.74_104.1073m/z
11.28_854.5921m/z
12.62_757.5610n
14.47_980.2530n
14.79_337.3338n
12.62_768.5867m/z
12.66_264.2447n
